# Sarcoidosis-like cutaneous lesions in paracoccidioidomycosis: four case reports and review of the literature^؆^

**DOI:** 10.1016/j.abd.2025.501250

**Published:** 2025-12-20

**Authors:** Jaqueline Santos Ribeiro, Rafael Fantelli Stelini, Renata Ferreira Magalhães, Paulo Eduardo Neves Ferreira Velho, Andrea Fernandes Eloy da Costa França

**Affiliations:** a Department of Internal Medicine, Faculty of Medical Sciences, Universidade Estadual de Campinas, Campinas, SP, Brazil; b Department of Pathology, Faculty of Medical Sciences, Universidade Estadual de Campinas, Campinas, SP, Brazil

*Dear Editor,*

Paracoccidioidomycosis (PCM) is a deep mycosis caused by the thermo-dimorphic fungi *Paracoccidioides brasiliensis* complex and *Paracoccidioides lutzii*. Although common in Latin America, its true prevalence is underestimated due to the lack of mandatory reporting. The clinical manifestations often develop many years after inhalation of conidia from soil due to the reactivation of endogenous latent foci.^1^

PCM can involve any organ, but its clinical presentation is typically classified into acute/subacute and chronic forms.^1^ Skin involvement occurs in up to 30% of patients with chronic PCM. The oral mucosa typically presents with ulcers featuring hemorrhagic dots (*moriform stomatitis*), whereas cutaneous lesions are polymorphic, predominantly ulcerative.^1^, ^2^

We describe four Brazilian patients with sarcoidosis-like skin lesions caused by PCM (Fig. 1, Fig. 2) and discuss the relevant literature. The female-to-male ratio was 3:1, with ages ranging from 25 to 51-years (average age of 38). The duration of skin lesions before diagnosis varied from 4-months to 4-years, and the most common misdiagnosis was leprosy (in 2 patients). The face was the first site involved in all patients, with systemic manifestations observed in the lungs (1 patient) and lymph nodes (2 patients). Skin biopsies showed granulomas with very few yeast elements, none exhibiting multi-sporulating characteristics. Culture and serology for Paracoccidioides sp. were positive in only one patient. All patients responded well to treatment with itraconazole or trimethoprim-sulfamethoxazole.

**Fig. 1:**
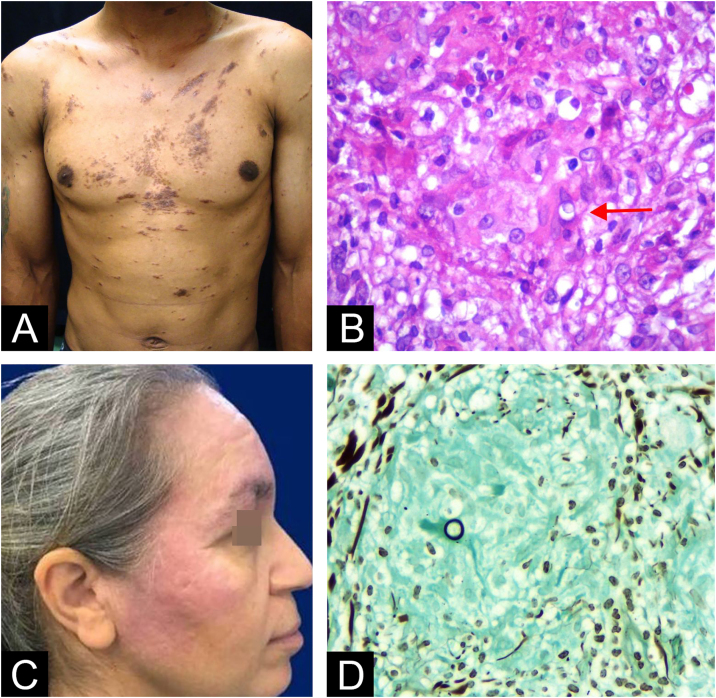
Patient 1 (A) Brownish plaques on the trunk and (B) A rare non-sporulating yeast element with thick wall in the dermis (arrow) at hematoxylin and eosin stain. Patient 2 (C) Red plaques on face and (D) Unique birefringent fungal structure within a dermal granuloma at Grocott stain.

**Fig. 2:**
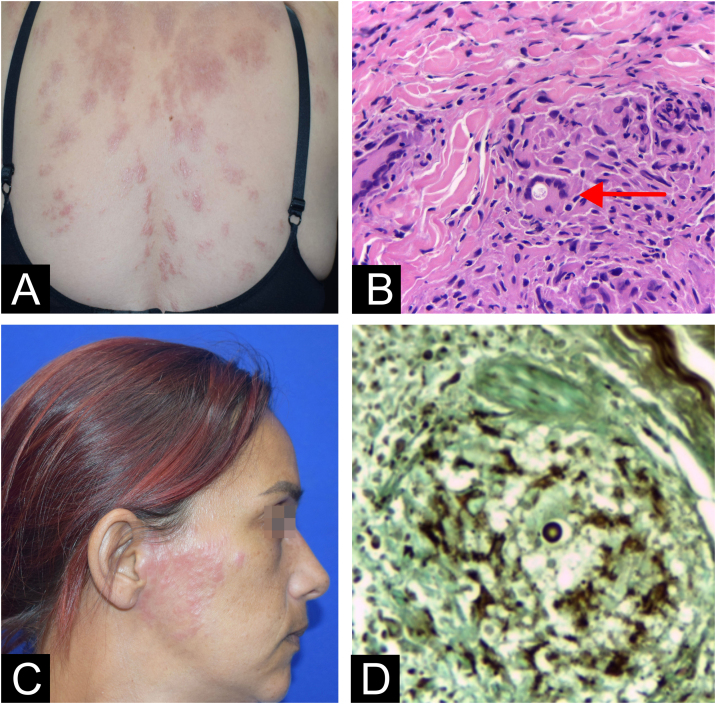
Patient 3 (A) Red plaques disseminated on the trunk and (B) Yeast inside giant cell (arrow) at hematoxylin eosin stain. Patient 4 (A) Pre-auricular red plaque and (D) Dermal granulomas showing an isolated yeast element at Grocott stain.

A literature search using the MeSH terms “*paracoccidioidomycosis*” AND “*sarcoidosis*” and “*paracoccidioidomycosis*” AND “*sarcoid*” in the PubMed and SciELO databases (August–September 2024) identified 12 articles describing sarcoidosis-like skin lesions in PCM. All publications were case reports or case series, dating from 2008 to 2024 (Table 1). A total of 13 patients were reported, primarily from the Southeast, South, and Central-West regions of Brazil.^3^, ^4^, ^5^, ^6^, ^7^, ^8^, ^9^, ^10^, ^11^, ^12^, ^13^, ^14^

**Table 1:** Summary of narrative review cases.

**Total of patients**	13
**Sex**	
Women	6
Men	7
**Age (average, in years)**	33
**Affected sites (%)**	
Head	100%
Trunk	30.8%
Limbs	7.7%
**Diagnostic delay (average, in years)**	2.8
**Lung involvement**	
Positive	0
Negative	10
Unknown	3
**PCM serology**	
Positive	4
Negative	3
Unknown	6
**Previous wrong diagnosis (%)**	
Granulomatous rosácea	5 (38%)
Leprosy	4 (30%)
Sarcoidosis	1 (7.6%)
Lupus erythematosus	1 (7.6%)
**Treatment**	
Itraconazole	7
Trimethoprim sulfamethoxazole	5
Amphotericin B	2
Unknown	2

Sarcoidosis-like cutaneous lesions in PCM present as brownish or reddish papules and plaques, which may or may not be infiltrated. The balance between immune defense and fungal infection determines the lesions' morphology and distribution, with the sarcoid-like form of PCM being associated with a predominant Th1 immune response. This presentation is typically paucifungal and classically resistant to the pathogen.^5^, ^15^ It seems that this immune response pattern is species independent.^10^, ^11^

Marques et al. analyzed 152 PCM patients treated at a Brazilian tertiary hospital and found that ulcerative and vegetative/verrucous lesions accounted for 50.1% of cases, whereas infiltrative lesions (including sarcoidosis-like forms) comprised 26.6% of cases. Although classic PCM lesions predominantly affect the cephalic region due to their contiguity with mucosal involvement, sarcoidosis-like PCM lesions also primarily affect this area, as observed in all cases from our study.^2^ The increased vascular perfusion in this region may further predispose to cephalic involvement.

Our review found that sarcoidosis-like PCM cases are more common in women, unlike the typical male predominance in other PCM forms. Estrogen receptors in *Paracoccidioides* spp. may inhibit the conversion of mycelium or conidia into the infectious yeast form, leading to fewer skin lesions and pulmonary involvement.^1^, ^5^ However, an extensive laboratory and imaging workup is recommended in all sarcoidosis-like PCM.^14^

Histologically, sarcoidosis-like PCM lesions are characterized by epithelioid granulomas with multinucleated giant cells, often containing few or no detectable fungal elements. This can lead to frequent misdiagnosis, as documented in 84% of the patients in our literature review. Sarcoidosis is the primary differential diagnosis, given its potential pulmonary and lymphatic involvement, as well as its cutaneous manifestations, which occur in 35% of cases.

Cutaneous sarcoidosis most commonly affects white women of working age, with papules and plaques frequently appearing on the face (61%) and often preceding systemic manifestations.

Leprosy, an endemic disease in Brazil, can histologically resemble PCM in its tuberculoid form. In ambiguous clinical presentations, histological findings may lead to misdiagnosis, resulting in inappropriate treatment and delayed correct diagnosis.^3^, ^6^, ^7^ To improve diagnostic accuracy, culture and serological testing ‒ preferably combining two modalities such as immunoblotting and immunodiffusion ‒ are recommended to rule out infectious causes.

Sarcoidosis-like PCM responds well to standard antifungal therapy, with rapid improvement of skin lesions. Nevertheless, treatment should be maintained for at least nine months. In cases where serologic tests are initially positive, they can be useful for monitoring treatment response.

## ORCID IDs

Jaqueline Santos Ribeiro: 0009-0009-4278-285X

Rafael Fantelli Stelini: 0000-0003-0618-1693

Renata Ferreira Magalhães: 0000-0001-9170-932X

Paulo Eduardo Neves Ferreira Velho: 0000-0001-7901-2351

## Authors' contributions

Jaqueline Santos Ribeiro: Writing of the manuscript; data collection.

Rafael Fantelli Stelini: Manuscript critical review; effective participation in the propaedeutics/therapeutics; final approval of the final version of the manuscript.

Renata Ferreira Magalhães: Manuscript critical review; final approval of the final version of the manuscript.

Paulo Eduardo Neves Ferreira Velho: Manuscript critical review; effective participation in the propaedeutics/therapeutics; final approval of the final version of the manuscript.

Andrea Fernandes Eloy da Costa França: Writing of the manuscript; effective participation in the research guidance; final approval of the final version of the manuscript.

## Financial support

This research did not receive any specific grant from funding agencies in the public, comercial, or not-for-profit sectors.

## Research data availability

Does not apply.

## Declaration of competing interest

None declared.
